# Novel Trends in Electrochemical Biosensors for Early Diagnosis of Alzheimer's Disease

**DOI:** 10.1155/2021/9984876

**Published:** 2021-09-01

**Authors:** Pavla Valkova, Miroslav Pohanka

**Affiliations:** Department of Molecular Pathology and Biology, Faculty of Military Health Science, University of Defense, Trebesska 1575, 50011 Hradec Kralove, Czech Republic

## Abstract

**Background:**

Alzheimer's disease (AD) is a multifactorial progressive and irreversible neurodegenerative disorder affecting mainly the population over 65 years of age. It is becoming a global health and socioeconomic problem, and the current number of patients reaching 30–50 million people will be three times higher over the next thirty years.

**Objective:**

Late diagnosis caused by decades of the asymptomatic phase and invasive and cost-demanding diagnosis are problems that make the whole situation worse. Electrochemical biosensors could be the right tool for less invasive and inexpensive early diagnosis helping to reduce spend sources— both money and time.

**Method:**

This review is a survey of the latest advances in the design of electrochemical biosensors for the early diagnosis of Alzheimer's disease. Biosensors are divided according to target biomarkers.

**Conclusion:**

Standard laboratory methodology could be improved by analyzing a combination of currently estimated markers along with neurotransmitters and genetic markers from blood samples, which make the test for AD diagnosis available to the wide public.

## 1. Introduction

Biosensors are simple but very precise analytical devices suitable for the detection of a broad spectrum of biological and chemical analytes. They are based on the conversion of a biological or chemical reaction into a measurable signal. Their application increases over years to medical, environmental, industrial, food, or pharmaceutical analysis mainly due to their fast response, accuracy, low cost, portability, and suitability for the point-of-care diagnosis [1, 2]. The history of biosensors dates back to the 1960s when Clark and Lyons presented the first biosensor. It was based on an oxygen electrode, so the first electrochemical biosensor was described [2, 3]. Many new biosensor designs are currently known. We can divide them either by their detection technique (called a physicochemical transducer) into magnetic, optical, electrochemical, mass-based, and thermal or by their biorecognition element, which is a biomolecule bound to the surface of the transducer. Enzymes, antibodies, genetic material (DNA, RNA), or whole cells and tissues are usually used as a biorecognition element. The biorecognition element provides a specific catalytic or binding reaction to the analyte, which is directly converted to a measurable signal by the transducer on its surface [4].

Alzheimer's disease (AD) is a neurodegenerative disorder characterized by an irreversible and progressive loss of selectively vulnerable populations of neurons [5, 6]. AD is an incurable devastating disorder that mainly affects the elderly population; it is currently estimated that more than 30 million people have AD, and their number is growing rapidly. Fast response to the first symptoms of AD is the key factor in the diagnosis of early stages of the disease, so treatment can be initiated and quality of life extended. Standard tests for AD, including magnetic resonance imagining, positron emission tomography (PET), near-infrared, or cerebrospinal fluid (CSF) analysis, are invasive and expensive and are usually performed on patients with the development of mild cognitive impairment. Thus, the low-cost test appropriate for the diagnosis of all stages of AD is required, and electrochemical biosensors represent a promising alternative for currently available diagnostic techniques [2, 7].

## 2. Electrochemical Biosensors

**Figure 1:**
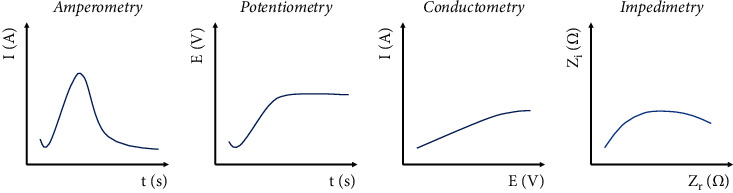
Division of electrochemical transducers according to the measured parameter and graphs of resulting curves.

Interest in electrochemical biosensors has increased over the years, mainly due to their high sensitivity, simple construction, easy handling, portability, low cost, ability to measure turbid samples, and compatibility, unlike the other types of biosensors [8, 9]. Electrochemical biosensors are based on the transformation of a biochemical signal into an amperometric signal while electrons are either generated or used. The amperometric signal is created by a potentiometric, amperometric, conductometric, or impedimetric transducer [3, 10]. The type of electrochemical sensor depends on the measured parameter: the electrical current is measured by the amperometric transducer, the generated potential is measured by the potentiometric transducer, conductance of medium is measured by the conductometric transducer, and impedance of medium is measured by impedimetric transducer [8]. Measured parameters of different electrochemical transducers are shown in Figure 1.

Amperometric biosensors are among the very simple detection techniques gaining interest in many scientific fields. The current resulting from the oxidation or reduction of electroactive substance is measured using a constant potential [11, 12]. Voltammetry is a method covered by amperometric techniques, but the varying potential is applied to the working electrode, and the change of current is observed [13]. Voltammetric techniques, including cyclic voltammetry, square wave voltammetry, differential pulse voltammetry, or linear sweep voltammetry, are often used in the construction of electrochemical biosensors [14]. Potentiometric biosensors are based on measuring potential/pH variation as a response to applied current, usually with low amplitude. It is often used in food, environmental, and clinical analysis to measure many organic and inorganic substances. The simplest potentiometric sensor is a pH or any other ion-selective electrode [15, 16]. Conductometric biosensors interpret a specific biological reaction on the transducer's surface as electrical conductance measured using a low-amplitude alternating electrical potential. The conductivity of sample change relies on the production or consumption of charged species [16, 17]. Impedimetric biosensors measure changes in impedance as a frequency function. Impedance is like resistance opposite of current flow, but resistance occurs in a direct current circuit, while impedance occurs in full alternating current circuits. Impedimetric biosensors usually determine affinity interactions between molecules [18, 19]. The pros and cons of different electrochemical techniques are summarized in Table 1.

**Figure 2:**
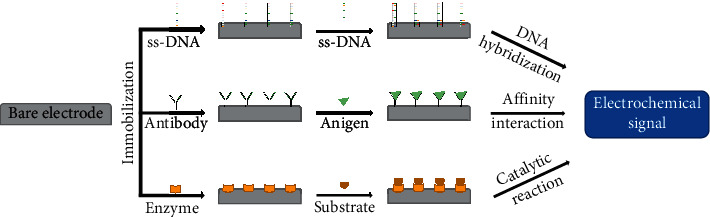
Biomolecules commonly used in the construction of electrochemical biosensors.

The electrochemical biosensors can also be divided into two groups regarding the electrochemical recognition process: biocatalytic sensors and bioaffinity sensors. The biocatalytic sensors are characterized by a catalytic reaction taking place on the surface of sensors and enzymes while the cells and tissues are appropriate biorecognition elements enabling this kind of reaction. On the other hand, bioaffinity sensors are typical for the affinity interaction taking place on the electrode surface, such as antigen-antibody interaction, nucleic acids interaction, or aptamer interaction. The bioaffinity biosensors with electrochemical detection are evolving rapidly because of their simplicity, low time, and high sensitivity, in contrast to traditional techniques for the detection of DNA, RNA, or antibodies such as polymerase chain reaction or fluorescence in situ hybridization. Their application in the diagnosis of AD is in great demand [7]. Biomolecules appropriate as biorecognition elements in electrochemical biosensors are shown in Figure 2.

## 3. Alzheimer's Disease

**Figure 3:**
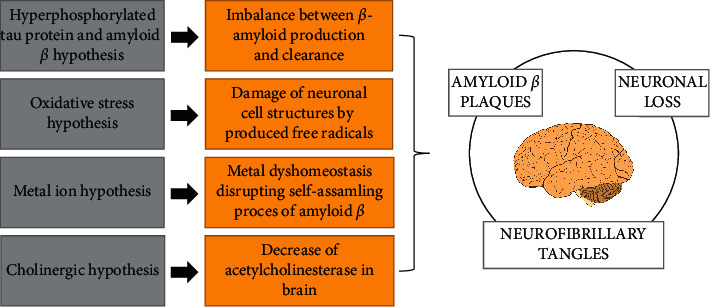
Hypothesis of AD pathology leading to A*β* plaques formation, formation of NFT of hyperphosphorylated tau protein, and neuronal loss.

AD is currently one of the most common forms of dementia. According to the World Health Organization, any type of dementia affects around 50 million of people worldwide and AD suffers to 60–70% of them [20]. According to the current estimates, the number of patients with AD will increase to more than 100 million by 2050. AD is thus becoming a global health and socioeconomic problem, due to the cost and time-demand of treatment [2, 5, 7]. AD characterized as progressive and irreversible decay of cognitive functions was first described in 1907 by German neurologist Alois Alzheimer. Typically, the memory and time and space orientation of older people are affected. People under the age of 65 years are susceptible rarely, only 4–6% of cases involve people younger than 65 years, and the early onset of AD is typically associated with genetic mutation [2, 21, 22]. Patients are affected by more dramatic multidomain cognitive impairment involving memory, attention, language, and visuospatial and executive behavior. The prodromal phase of AD dementia rarely precedes, and a faster progression to a severe condition is observed [23]. AD is a multifactorial disease characterized by extracellular deposition of amyloid *β* (A*β*) peptides forming senile plaques by intraneuronal neurofibrillary tangles (NFT) of hyperphosphorylated tau protein and substantial synaptic and neuronal loss. Amyloid angiopathy is also common. The hippocampus, amygdala, entorhinal cortex, and cortical association areas belong to the most affected brain areas [2, 6, 24]. Aggregation of A*β* is possibly accelerated by chelation with metal ion mainly Cu^II^ and Fe^III^ [25, 26]. The etiology of AD is still unclear, and the probable pathological thesis is summarized in Figure 3 [24, 27, 28].

The first changes in the brain begin years, even decades, before symptoms appear. This phase is called the pathophysiological or clinical stage, and it is characterized as an asymptomatic period no different from normal aging. A*β* is deposited in neocortical areas of the brain without cognitive or behavioral changes. On the other hand, it is still not clear whether all A*β* deposition leads to the development of AD. The diagnostic methodology for this phase has not been established. The National Institute on Aging and the Alzheimer's Association focused on the design of diagnostic criteria and biomarkers appropriate for the diagnosis of early AD stages [5, 7, 29, 30]. The prodromal stage of AD known as mild cognitive impairment (MCI) is the period between normal aging and AD, and patients are cognitively impaired, usually in memory but not demented. Cognitive functions such as orientation, language, attention, and executive function may also be affected, and patients may have mild problems to easily perform common daily tasks. Patients suffering MCI have an increased risk of developing AD, so MCI treatment aims to reduce this risk [5, 31–33]. The third phase, called AD dementia, is characterized as a slow progression of memory disturbance followed by other cognitive domains' disturbance (language, social, and occupational dysfunctions) and behavioral changes. Plaques of deposited A*β* and NFT are the main pathological features of this stage [5, 31]. Three neuroimaging markers were established for the diagnosis of Alzheimer's disease: magnetic resonance imagining (MRI) of hippocampal atrophy, fluorodeoxyglucose PET (FDG-PET) of temporoparietal hypometabolism, and PET of increased brain deposition of A*β* and NFT [23].

The individual phases are overviewed in Table 2.

## 4. Alzheimer's Disease Biomarkers

### 4.1. CSF Biomarkers

**Figure 4:**
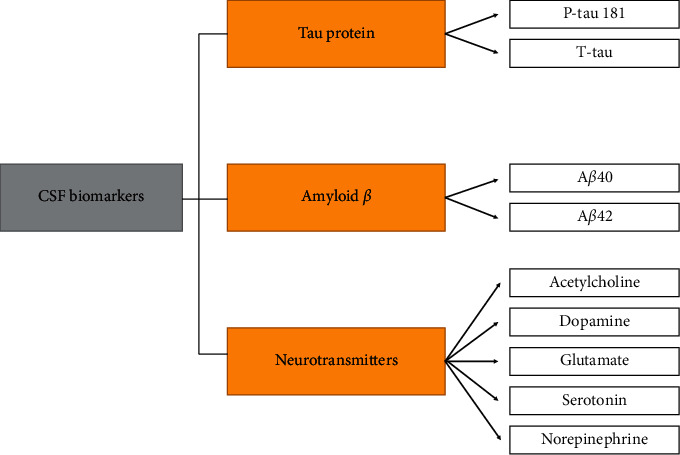
Overview of the most common CSF biomarkers.

The analysis of CSF biomarkers, reliable indicators of cerebral neurochemistry, is an established diagnostic method achieving high specificity in the diagnosis of AD (survey in Figure 4). CSF biomarkers are perfect as diagnostic criteria for the early stages of AD. On the other hand, obtaining CSF is an invasive and painful procedure with possible side effects, especially in the elderly. CSF analysis is performed either immunohistochemically or by enzyme-linked immunosorbent assay. Both methods are relatively expensive and time consuming. To date, three biomarkers have been established for the diagnosis of AD from CSF: phosphorylated tau protein (P-tau) 181, total tau protein (T-tau), and A*β* [2, 7, 10]. Decreased A*β*_42_ alone or in combination with A*β*_40_ and increased P-tau_181_ and T-tau is a typical profile for the diagnosis of AD from CSF [34]. The analysis of these three CSF biomarkers has high diagnostic accuracy (85–90%) in the diagnosis of AD and also in the diagnosis of MCI, so they have been incorporated in the diagnostic criteria of AD [35].

#### 4.1.1. P-tau_181_ and T-tau

Tau protein is a microtubule-associated protein that normally stabilizes microtubules in the cell cytoskeleton, but it is also forming intracellular aggregates in several neurodegenerative diseases including AD. Phosphorylation of tau protein is a physiological process ensuring regulation of tau protein production. Under pathological conditions, the tau protein becomes hyperphosphorylated and causes protein separation from the microtubules and destabilizes them structurally, so axonal transport is disrupted. Hyperphosphorylated tau protein forms masses of paired helical filaments inside the nerve cell bodies—the NFT, one of the clinical criteria for AD diagnosis [10, 36, 37]. Thus, tau protein has become a biomarker evaluated in the AD diagnosis as a sign of axonal degeneration and NFT formation. However, the tau protein exists in the human brain in 6 isoforms produced from a single gene that differ in posttranslational modification including phosphorylation, glycosylation, and oxidation. The tau protein isoform phosphorylated on threonine 181 (P-tau_181_) is important for AD diagnosis. While the P-tau_181_ may be a more specific marker of AD, general T-tau belongs to general CSF markers of all neurodegeneration, so, elevated levels of T-tau may be also detected in other tauopathies. This is due to the NFT structure, typical for AD, which consists of hyperphosphorylated tau protein [10, 34, 37, 38].

#### 4.1.2. A*β*_42_ and A*β*_40_

Any imbalance in the production or clearance of amyloidogenic A*β* peptides leads to extraneuronal accumulation of A*β* in amyloid plaques, the second well-documented pathology of AD, and also the second clinical diagnostic criteria of AD. A*β* peptides, normally soluble, exist in many conformations consisting of 36–43 amino acids, so they are labeled according to the length of their amino acid chain. In particular, A*β*_42_ most easily succumb to aggregation into A*β* plaques. A*β*_42_ and A*β*_40_ are the most reliable indicators of AD. Compared to the healthy population, the analysis of A*β*_42_ from CSF in AD patients shows a substantial decrease, probably caused by its aggregation into plaques. On the other hand, A*β*_40_ CSF levels do not correlate with AD status, the levels are not decreased, and it is not formed in amyloid plaques. Despite this fact, its levels are measured in the diagnosis of AD, given that the ratio A*β*_42_/A*β*_40_ shows a significant decrease. The analysis of ratio A*β*_42_/A*β*_40_ has a more corresponding value than the measurement of A*β*_42_ alone, as it compensates for intraindividual changes within AD patients and has a better association with the pathogenesis of AD [7, 30, 36].

#### 4.1.3. Neurotransmitters

Neurotransmitters are endogenous chemical compounds that act as communication messengers between neurons. To date, more than a hundred neurotransmitters have been discovered [9]. AD is not a disease affecting only the cognitive functions of the brain, but noncognitive symptoms have been observed, including agitation, anxiety, depression, apathy, psychosis, and sleep or appetite disorders. Various studies suggested that neurotransmitters have a key role in both cognitive and behavioral dysfunctions of AD [39]. Neurotransmitters including acetylcholine, dopamine, serotonin, glutamate, and norepinephrine have been studied as factors influencing the development of neurodegeneration and as possible biomarkers of early stages of AD [7, 40].

### 4.2. Blood Biomarkers

**Figure 5:**
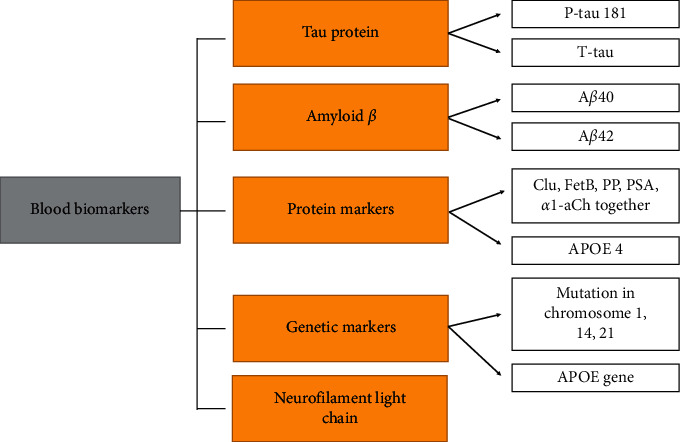
Overview of the most promising blood biomarkers. Clu: clusterin; FetB: fetuin B; PP: pancreatic prohormone; PSA: prostate-specific antigen; *α*1-aCh: *α*1-antichymotrypsin.

So far, the analysis of CSF and neuroimaging, mainly PET scan, has the dominant role in AD diagnosis as well as in AD drug design. Although the analysis of biomarkers from CSF in the diagnosis of AD is a specific and established method with high diagnostic accuracy, it is still an invasive intervention in the body. In recent years, therefore, there has been a growing tendency to establish a methodology for the diagnosis of AD from plasma samples [2, 41]. Possible problems when using blood samples to measure AD biomarkers could be high interferences and the ability of the biomarker to penetrate from CSF to blood through the blood brain barrier [30]. The following biomarkers described below including APOE 4, protein p53, and the other proteins are very often also CSF biomarkers (8 Toyos-Rodriguez 2020). A survey of the most promising blood biomarkers is given in Figure 5.

#### 4.2.1. A*β* and Tau

Initial studies did not suggest a relevant correlation between CSF and blood levels of A*β*_42_ or ratio A*β*_42_/A*β*_40_, as was summarized in the meta-analysis of Olsson and coauthors (2016) [35]. On the other hand, new studies showed promising results in the analysis of A*β* from blood. In the work of Janelidze and coauthors (2016), a weak positive correlation between CSF and blood levels for both A*β*_42_ and A*β*_40_ was observed mainly in patients with AD dementia. Prodromal stages showed just a moderate decrease in A*β*_42_ levels and no decrease in A*β*_40_ levels. This work reveals that plasma changes of A*β* levels occur much later in comparison with the levels of A*β* in CSF [42]. The possibility of A*β* measurement was also studied in the work of Ovod and coauthors (2017) and Nakamura and coauthors (2018) with a proven correlation between A*β* levels in the blood and CSF [41, 43, 44]. Regarding the measurement of plasma T-tau and P-tau, the work of Toombs and Zetterger (2020) mentioned a contrast result from various studies. It probably depends on the sensitivity of the chosen method. Using ultrasensitive assays, plasma tau levels were increased compared to the healthy population, but this increase is negligible in comparison with levels of tau from CSF. On the other hand, increased plasma levels of P-tau_181_ correlate perfectly with increased CSF levels of P-tau_181_ [41, 45].

#### 4.2.2. Neurofilament Light Chain

The neurofilament light chain is an intraaxonal structural protein that leaks into body fluids, both CSF and blood, during axonal damage, regardless of the cause. Although it is not clear how blood levels of NFL correlate with neurodegeneration, it belongs to the most consistent plasma biomarkers of neurodegeneration [41, 45].

#### 4.2.3. Protein Markers

Plasma levels of proteins including clusterin, fetuin B, pancreatic prohormone, and prostate-specific antigen complexed to *α*1-antichymotrypsin are altered during AD and can be analyzed as good biomarkers but only together represent a reliable tool for diagnosis with strong correlation to AD [2]. Apolipoprotein E4 (APOE4) is considered a risk factor for the development of AD and has been introduced as a plasma biomarker for early diagnosis of AD. APOE is a glycoprotein that mediates the binding of lipoproteins to their low-density receptors. They are predominantly expressed in the brain in several isoforms, APOE2, APOE3, and APOE4 belong to the most common ones, but only APOE4 presents a risk factor to AD by reducing amyloid clearance and accelerating senile plaques formation. Circa 50% of all AD patients have APOE4 isoform [7, 10]. The protein p53 responds to cellular stress, and the relationship between conformationally altered p53 and AD diagnosis in blood has been studied since 2008 when Lanni and coauthors observed unfold p53 in peripheral blood cells of AD patients. Recently, unfolded protein p53 was determined from blood in the work of Amor-Gutiérrez and coauthors (2020) using a competitive electrochemical immunosensor with promising results [46, 47].

#### 4.2.4. Genetic Markers

The AD, not only early-onset type, can be diagnosed by the analysis of the genome of patients. Genetic mutations and polymorphisms related to the disease can be discovered. Single gene mutation of chromosomes 1, 14, and 21 causes malformations on amyloid precursor protein, presenilin 1, and presenilin 2, and it is the cause of the early onset of AD. The above-mentioned polymorphism of APOE is the probable most common genetic cause of late onset of AD and can be also revealed by the analysis of the genetic material [2].

#### 4.2.5. Metals

Metals, including Cu^II^, Zn^II^, and Fe^III^, may play an important role in AD pathology due to their high concentration in senile plaques. Mainly redox-active metals (Cu and Fe) are able to bound on A*β*, stabilize its oligomeric form, and accelerate the aggregation of A*β* [25, 26]. Recently, altered plasma metal levels were observed in conditions involving CNS-associated disorders in the work of Nahan and coauthors (2017). According to the work of Xu and coauthors (2018) and Guan and coauthors (2017), plasma metals are suggested as potential blood markers [48–50].

## 5. Electrochemical Biosensors in Diagnosis of Alzheimer's Disease

### 5.1. Detection of A*β*

In recent years, highly sensitive electrochemical biosensors have been designed for the detection of A*β*, especially A*β*_42_ and A*β* oligomers. With respect to the type of biorecognition element, three main groups of structures were used: RNA aptamer, antibody, and molecularly imprinted polymers (MIP). The developed biosensor devices are typically portable and simple tools commonly suitable for point-of-care use when a commercial product based on the research will be introduced into the market.

In the work of Negahdary and Heli (2019), RNA aptamer was immobilized onto the surface of gold disk electrode with electrodeposited fern leaves-like gold nanostructure intended to detect A*β*_42_ [51].

MIP has also been used in the work of Ozcan and coauthors (2020) and Pereira and coauthors (2020), both aimed to detect A*β*_42_ [52, 53]. The classic electrochemical biosensor construction, a glassy carbon electrode with delaminated titanium carbide MXene and multiwalled carbon nanotubes composite covered by MIP, was prepared in the work of Ozcan and coauthors [52]. On the other hand, in the work of Pereira and coauthors, an innovative paper-based platform for carrying MIP was prepared [53]. Although Ozcan and coauthors have achieved a much lower limit of detection as well as better sensitivity, the construction of Pereira and coauthor is unique in its simplicity and low cost.

The most widely used biorecognition elements were antibodies. In the work of Le and coauthors (2020), a self-assembled monolayer functionalized interdigitated chain-shaped electrode with immobilized specific anti-A*β* antibody was prepared for the detection of A*β*_42_ and used together with atypical nonfaradaic detection [54]. The self-assembled monolayer was also used in the construction of biosensors in the work of Carneiro and coauthors (2017). They modified the gold electrode by mercaptopropionic acid SAM, gold nanoparticles, and monoclonal antibody mAb DE2B4 for the analysis of A*β*_42_ [55]. Sethi and coauthors (2020) used a screen-printed electrode with a dual layer of graphene-reduce graphene oxide to immobilize the H31L21 antibody. The sensor was designed for use in the rapid detection of A*β*_42_ [56].

A unique biorecognition element-cellular prion protein was used in the work of Qin and coauthors (2020). A gold electrode with immobilized gold dendrite and electropolymerized poly(pyrrole-3-carboxylic acid) was used as a carrier for this bioreceptor for the detection of A*β* oligomers [57]. A comparison of the detection limits, linear concentration ranges, or detection techniques of biosensors described above is shown in Table 3.

### 5.2. Detection of tau Protein

Recent approaches in electrochemical biosensors for tau protein detection were based on antibody biorecognition elements. Different types of tau isoforms are known. Tau-441 was detected in the work of Carlin and Martic-Milne (2018), Karaboga and Sezginturk (2020), and Wang and coauthors (2017) [58–60]. In the first-mentioned paper, a very simple constriction of gold electrode covered by anti-tau antibodies was designed [58]. The paper of Karaboga and Sezginturk introduced a more complicated construction of indium tin oxide electrode coated by polyethylene terephthalate and utilizing nanocomposite of reduced graphene oxide and gold nanoparticles for antibodies binding [59]. Wang and coauthors prepared a four-electrode system of gold microband electrodes covered with a layer of a self-assembled monolayer and protein G. Protein G is used to interact with immobilized antibodies to ensure their optimal orientation [60]. The fact is that the use of a nanocomposite structure in biosensor construction highly increases the sensitivity of the sensor, according to the available limit of detections.

The tau-381 isoform was determined in the work of Shui and coauthors (2018). In this paper, a combination of antibodies and aptamer as biorecognition elements was used in Sandwich assay construction. The gold working electrode was used as a carrier of cysteamine-stabilized AuNPs covered by biorecognition elements [61].

T-tau protein was determined by a gold electrode coated with a self-assembled monolayer of 3-mercaptopropionic acid with immobilized anti-T-tau antibodies in the work of Dai and coauthors (2017) and a screen-printed carbon electrode with gold nanoparticles-poly(amidoamine) dendrimer nanocomposite and anti-tau capture antibody for tau protein detection was designed in work of Razzino and coauthors (2020) [62, 63]. Further details on the described electrochemical biosensors for the detection of tau protein are summarized in Table 4.

### 5.3. Detection of Neurotransmitters

Chemical compounds that serve as biological messengers from a nerve cell through a synapse to a target cell are called neurotransmitters. In recent years, various neurotransmitters have been measured for early diagnosis of AD.

Electrochemical biosensors for acetylcholine detection were designed by da Silva and Brett (2020), Chauhan and coauthors (2017), and Moreira and coauthors (2017), and all of these sensors contained the enzyme acetylcholinesterase as biorecognition element [64–66]. The most sensitive one, glass plate modified by iron oxide nanoparticles-poly(3,4-ethylenedioxythiophene)-reduced graphene oxide nanocomposite with immobilized enzymes acetylcholinesterase and choline oxidase, was constructed by the team of Chauhan and coauthors [65]. Moreira and coauthors prepared a biosensor based on platinum wire covered by a highly porous gold film and enzyme acetylcholinesterase, and it shows the lowest limit of detection [66]. The biosensor prepared by da Silva and Brett was based on a glassy carbon electrode modified by iron oxide nanoparticles covered by a film of poly(neural red), and acetylcholinesterase was immobilized onto its surface [64].

Another neurotransmitter, dopamine, was the target of a biosensor construction designed by Shin and coauthors (2017) and Yi and coauthors (2017). Both biosensors were enzymeless, the first one based on indium tin oxide electrode covered by graphene oxide and silver nanoparticles, and the second one based on self-supporting nanoporous gold wire with palladium nanoparticles [67, 68]. Both sensors showed similar detection limits and so similar sensitivity.

The papers of Hughes and coauthors (2015) and Alves and coauthors (2016) were focused on the determination of glutamate by electrochemical biosensors [69, 70]. The enzymatic sensor was based on a screen-printed carbon electrode covered by a layer of chitosan with multiwalled carbon nanotubes and the enzyme glutamate dehydrogenase and its cofactor nicotinamide adenine dinucleotide [69]. The nonenzymatic sensor was based on a graphite electrode with an immobilized small protein-like chain designed to mimic a peptide that recognizes glutamate [70]. Electrochemical biosensors for the detection of neurotransmitters are listed in Table 5.

### 5.4. Detection of Genetic and Protein Markers

MicroRNA has become a highly determined biomarker of AD. Four works focusing on the detection of microRNA-137, microRNA-146a, and microRNA-34a are described lower. Azimzadeh and coauthors (2017) designed an electrochemical biosensor for the detection of microRNA-137 based on screen-printed carbon electrodes modified by reduced graphene oxide-gold nanowire nanocomposite, and doxorubicin was immobilized as an intercalated label [71]. A gold electrode with self-assembled capture microRNA for bioconjugation with microRNA-146a was constructed by Khalilzadeh and coauthors (2019) [72]. Detection of microRNA-34a was the aim of two studies performed by two teams from Ege University. An older study described the preparation of pencil graphite electrodes with a DNA probe for microRNA-34a [73]. The second article described impedimetric biosensor based on screen-printed carbon electrode with immobilized 3.5 poly(amidoamine) dendrimer and DNA probe for microRNA-34a, and it showed better sensitivity than voltammetric biosensor [74].

APOE is encoded by a gene having three isoforms (E2, E3, and E4) [75]. APOE4 is considered a risk factor for the development of AD [7]. Mutations of APOE can be detected by genosensors, and the protein itself can be detected mainly by immunosensors. Jafari and coauthors (2019) designed genosensor based on a glassy carbon electrode with an immobilized reduced graphene oxide-cerium oxide nanoparticles nanocomposite modified by ssDNA probe for APOE gene, namely, for 23-base oligonucleotide sequences with a point mutation [75]. On the other hand, Liu and coauthors (2020) prepared an immunosensor based on a glassy carbon electrode with gold nanobipyramid coated platinum nanostructure coated by anti-APOE4 antibody and gold : palladium-polydopamine nanotube nanozyme [76].

An electrochemical biosensor for another protein biomarker of AD, clusterin, was constructed in the work of Islam and coauthors (2018). Label-free immunosensor was based on a screen-printed carbon electrode with anti-clusterin antibody fragments [77].

Electrochemical immunosensors for the detection of conformationally altered (unfolded) protein p53 have also been proposed for the early diagnosis of AD. A simple screen-printed carbon electrode with bound anti-p53 antibody was prepared by Tonello and coauthors (2016), but only preliminary results were described [78]. Iglesias-Mayor and coauthors (2020) designed an immunosensor based on bifunctional core-shell Au–Pt/Au and immobilized anti-p53 antibody. This proposal was based on competition between protein p53 in the sample and p53 bounded to streptavidin modified magnetic beads added in measured solution, so, the higher concentration of p53 in the sample caused the lower catalytic current response [79]. The electrochemical geno- and immunosensors described above are summarized in Table 6.

## 6. Perspectives in the Diagnosis of Alzheimer's Disease

Because the number of AD patients is growing rapidly and no therapeutic drug to cure AD was found, early diagnosis is the key factor in managing and slowing the disease. The most important step for the future diagnosis of AD lies in early diagnosis before a severe clinical symptom appears. Preventive testing should include biomarkers that precede these symptoms [10, 80]. This examination should be noninvasive, affordable, simple, and fast. Less invasive analysis of blood biomarkers is a promising possibility, but noninvasive analysis of urine, tears, sweat, or saliva would overcome the limitation of invasive sampling [80, 81]. AD as a multifactorial disease with still unclear pathology is very hard to diagnose. Biosensors for simultaneous detection of multiple biomarkers would simplify and speed up diagnostics from just one sample. So, the important strategy is to integrate more analytical technologies into one platform. The use of nanotechnologies would provide high sensitivity and specificity of biosensors providing analysis of very low concentrations of biomarkers in noninvasively taken samples [81, 82]. Molecularly imprinted polymers allow the analysis of high-affinity aptamers and antibodies specific for AD biomarkers. This is an important challenge for the development of biosensors, which should pay close attention to this problem [10, 82].

Electrochemical biosensors are technology growing rapidly in the diagnosis of AD, but the extension of tested biomarkers would facilitate their incorporation into clinical practice. Many biomarkers including metal ions or posttranslational protein modifications are still neglected. Heavy metals have been analyzed by electrochemical biosensor from water in the work of Sciuto and coauthors (2020), copper from water samples was determined in the work of Cui and coauthors (2014), and iron from the water was tested in the work of Kamal and coauthors (2014) [83–85]. Glycan electrochemical biosensor for cancer diagnosis was presented in the work of Kveton and coauthors (2019), the electrochemical behavior of phosphotyrosine was observed in the work of Popa and Duculescu (2013), and dual-mode sensor using an electrolyte–insulator–semiconductor field-effect device coupled with nanoplasmonic effects was developed for protein phosphorylation detection in the work of Bhalla and coauthors (2015) [86–88]. So, various electrochemical biosensors exist, but their optimization and application in AD diagnosis were not tried yet.

## 7. Conclusion

AD is a serious health and socioeconomic problem, and its solution is mainly in early and simple but precise diagnosis, so proper diagnostic techniques are required. Simple sensors suitable for point-of-care use are especially desired. Electrochemical biosensors are the future of AD diagnostics due to their high sensitivity, simple construction, easy handling, portability, and low cost. Although many possible diagnostic methods have recently been investigated, their integration into clinical practice and diagnostic protocol is necessary, especially since early diagnosis of AD is a crucial part of good treatment and patient's state outcome. Detection of blood biomarkers instead of CSF biomarkers is one of the key parts of this diagnostic protocol which could also make the diagnosis more accessible to all patients with or without AD symptoms. Although electrochemical biosensors for the detection of A*β*, tau proteins, neurotransmitters, and genetic and protein markers have been discussed in this review, laboratory determination of A*β*42 alone or in combination with A*β*40, P-tau_181_, and T-tau has been established into practice so far. Standard laboratory methodology could be improved by analyzing a combination of the currently estimated markers along with neurotransmitters and genetic markers from blood samples, which make the test for AD diagnosis available to the wide public. This protocol could detect AD in asymptomatic patients and prolong their quality lifetime.

## Figures and Tables

**Table 1:** Summary of advantages and disadvantages of various electrochemical techniques.

Electrochemical technique	Advantages	Disadvantages
Amperometry	Fastness, sensitivity, precision	Poor selectivity
Potentiometry	Measuring of low concentration and turbid samples	Possibility of false-negative result by strong buffers
Conductometry	Use in living biological system	Low specificity
	No reference electrode needed easy miniaturization; they measure both electroactive and inactive analytes	
Impedimetry	Possibility of label-free setup; they can be applied in living biological system and they exert high sensitivity and suitability for miniaturization	Poor selectivity

**Table 2:** Overview of AD phases [5, 10, 30–32].

Phase	Symptoms	Neuroimaging	Biomarkers
Clinical	None	None	None
MCI	Change of cognitive ability	MRI, PET, FDG-PET	CSF levels of T-tau, P-tau, and A*β*_42_
	Impairment of at least one cognitive domain		
	Mild problems in performing complex tasks not demented		
AD dementia	Cognitive and behavioral dysfunctions	MRI, PET, FDG-PET	CSF levels of T-tau, P-tau, and A*β*_42_

**Table 3:** Overview of electrochemical biosensors for the detection of A*β* in AD diagnosis.

Marker	Construction	LOD/pM	Liner range/pM	Detection	References
A*β*_42_	Fern leaves-like gold nanostructure with an RNA aptamer	88.6 10^−3^	0.440–285	DPV	[51]
A*β*O	PPy-3-COOH electropolymerized onto gold dendrite with bounded cellular prion protein	1 10^−6^	10^−6^–10 10^3^	Impedimetry	[57]
A*β*_42_	SAM functionalized interdigitated chain-shaped electrode with anti-A*β* antibody	1.70	2.20–2.20 10^3^	Nonfaradaic detection	[54]
A*β*_42_	GCE with titanium carbide MXene and MWCN composite including MIP	6.65 10^−5^	2.20 10^−4^–2.20 10^−2^	DPV	[52]
A*β*_42_	Dual layer of graphene and rGO with immobilized H31L21 antibody achieved via Pyr-NHS	2.40	11.0–55.0 10^3^	DPV	[56]
A*β*_42_	MIP in a paper-based platform on the carbon ink electrode's surface	14.8	22.0–22.0 10^4^	SWV	[53]
A*β*_42_	Gold electrode with mercaptopropionic acid SAM, gold nanoparticles, and monoclonal antibody mAb DE2B4	1.15	2.20–2.20 10^2^	SWV	[55]

LOD: limit of detection, DPV: differential pulse voltammetry, A*β*O: A*β* oligomers, PPy-3-COOH: poly(pyrrole-3-carboxylic acid), SAM: self-assembled monolayer, GCE: glassy carbon electrode, MWCN: multiwalled carbon nanotubes, MIP: molecularly imprinted polymers, rGO: reduced graphene oxide, Pyr-NHS: 1-pyrenebutyric acid N-hydroxysuccinimide ester, and SWV: square wave voltammetry.

**Table 4:** Overview of electrochemical biosensors for tau protein detection in AD diagnosis.

Marker	Construction	LOD/pM	Liner range/pM	Detection	Reference
Tau-441	Anti-tau antibodies immobilized onto a gold electrode	10^6^–10^3^	–	CV, SWV	[58]
T-tau	SAM of MPA binding anti-T-tau antibody on the gold electrode	–	–	DPV	[62]
Tau-381	Cysteamine-stabilized AuNPs with anti-tau antibody and an aptamer specific to tau-381	0.420	0.500–1.00 10^2^	DPV	[61]
Tau	SPCE modified with an AuNPs-PAMAM dendrimer nanocomposite and anti-tau capture antibody	0.030	0.110–91.0	Amperometry	[63]
Tau-441	ITO-coated PET electrode with rGO-AuNPs nanocomposite and anti-tau antibodies	0.002	2.20 10^−2^–10.9	EIS, CV	[59]
Tau-441 (2N4R)	Four gold microband electrodes with a layer of a SAM, protein G, and anti-tau antibodies	0.030	–	CV, EIS	[60]

LOD: limit of detection, CV: cyclic voltammetry, SWV: square wave voltammetry, SAM: self-assembled monolayer, MPA: 3-mercaptopropionic acid, DPV: differential pulse voltammetry, AuNPs: gold nanoparticles, SPCEs: screen-printed carbon electrode, PAMAM: poly(amidoamine), rGO: reduced graphene oxide, ITO: indium tin oxide, PET: polyethylene terephthalate, and EIS: electrochemical impedance spectroscopy.

**Table 5:** Overview of electrochemical biosensors for the detection of neurotransmitters in AD diagnosis.

Marker	Construction	LOD/*μ*M	Liner range/*μ*M	Detection	Reference
ACh	GCE modified by IONPs with poly(neutral red) film and AChE	1.00	2.50–60.0	Amperometry	[64]
ACh	Glass plate with IONPs-PEDOT-rGO nanocomposite modified by FTO and immobilized AChE and ChO	4.00 10^−3^	4.00 10^−3^–8.00 10^2^	CV	[65]
ACh	Pt wire covered by highly porous gold film with immobilized AChE	10.0	0.250 10^3^–1.90 10^3^ (PBS) 0.120 10^3^–1.40 10^3^ (GB)	LSV, SWV, CA	[66]
Dopamine	ITO electrode covered by GO and SNPs	0.200	0.100–1.00 10^2^	CV, DPV, amperometry	[67]
Dopamine	Self-supporting NPG wire with PdNPs	Up to 1.00	1–2.20 10^2^	DPV	[68]
Glutamate	SPCE with chitosan and MWCN encapsulating GLDH and NAD^+^	3.00	7.50–105	Amperometry	[69]
Glutamate	Graphite electrode with mimetic peptide recognizing glutamate	0.001	1.00 10^3^–10.0 10^3^	DPV	[70]

LOD: limit of detection, ACh: acetylcholine, GCE: glassy carbon electrode, IONPs: iron oxide (Fe2O3) nanoparticles, AChE: acetylcholinesterase, PEDOT: poly(3,4-ethylenedioxythiophene), rGO: reduced graphene oxide, FTO: fluorine-doped tin oxide, ChO: choline oxidase, CV: cyclic voltammetry, PBS: phosphate-buffered solution, GB: glycine buffer, LSV: linear sweep voltammetry, SWV: square wave voltammetry, CA: chronoamperometry, ITO: indium tin oxide, GO: graphene oxide, SNPs: silver nanoparticles, DPV: differential pulse voltammetry, NPG: nanoporous gold, PdNPs: palladium nanoparticles, SPCE: screen-printed carbon electrode, MWCNTs: multiwalled carbon nanotubes, GLDH: glutamate dehydrogenase, and NAD+: nicotinamide adenine dinucleotide.

**Table 6:** Overview of electrochemical biosensors for the detection of genetic and protein markers in AD diagnosis.

Marker	Construction	LOD/nM	Liner range/nM	Detection	Reference
miR-137	SPCE with rGO and Au nanowires and label doxorubicin	1.70 10^−6^	5.00 10^−6^–0.750 10^−3^	DPV	[71]
miR-146a	Au electrode modified by capture miR	10.0 10^−3^	10.0 10^−3^–1.00 10^3^	SWV	[72]
miR-34a	SPCE with PAMAM dendrimer and DNA probe for miR-34a	140	0–10.7 10^2^	Impedimetry	[74]
miR-34a	PGE with GO and miRNA-34a specific DNA probe	10.7 10^2^	7.10 10^2^–5.00 10^3^	DPV	[73]
APOE gene	GCE electrode modified by rGO-CONPs nanocomposite with ssDNA probe for APOE gene	1.00 10^−6^	10.0 10^−6^–10.0	SWV	[75]
APOE4	GCE modified by Au nanobipyramid coated Pt nanocomposite with anti-APOE4 antibody and AuPd-PDA nanozyme	0.450 10^−3^	1.50 10^−3^–58.0	Amperometry	[76]
Clusterin	SPCE with anti-clusterin antibody fragments	Down to 3.20 10^−5^	3.20 10^−5^–3.20 10^−3^	CV, SWV	[77]
p53	SPCE with anti-p53 antibody	—	—	ASV	[78]
p53	SPCE modified by bifunctional core-shell Au–Pt/Au NPs with anti-p53 monoclonal antibody	66.0	—	CA	[79]

## Data Availability

All the data are inside the manuscript.

## Abbreviations

AD: Alzheimer's disease
A*β*: Amyloid *β*
CSF: Cerebrospinal fluid
FDG-PET: Fluorodeoxyglucose positron emission tomography
MRI: Magnetic resonance imagining
NFT: Neurofibrillary tangles
PET: Positron emission tomography
P-tau: Phosphorylated tau protein
T-tau: Total tau protein.
